# Outcomes of endoscopic submucosal dissection for treatment of superficial anal squamous cell carcinoma: Multicenter international experience

**DOI:** 10.1055/a-2641-5597

**Published:** 2025-07-24

**Authors:** Miguel Fraile-López, Mathieu Pioche, Jérôme Rivory, Anastassios Manolakis, Yoji Takeuchi, Shiko Kuribayashi, Keigo Sato, Alberto Herreros de Tejada, Diego de Frutos Rosa, Takashi Kanesaka, Mayo Tanabe, Amyn Haji, João Santos-Antunes, Rui Morais, Zacharias Tsiamoulos, Hugo Uchima, Adolfo Parra-Blanco, Maria Luisa Cagigal, Alvaro Terán, Enrique Rodriguez de Santiago

**Affiliations:** 1 Gastroenterology and Hepatology Department, Clinical and Translational Research in Digestive Diseases, Valdecilla Research Institute (IDIVAL), Marqués de Valdecilla University Hospital, Santander, Spain; 2 Gastroenterology and Endoscopy Unit, Edouard Herriot Hospital, Lyon, France; 3 69176 Department of Gastroenterology, General University Hospital of Larissa, Larissa, Greece; 4 41678 Endoscopy and Endoscopic Surgery, Gunma University Hospital, Meabashi, Japan; 5 53312 Department of Gastrointestinal Oncology, Osaka International Cancer Institute, Osaka, Japan; 6 Department of Gastroenterology and Hepatology, Gumma University Graduate School of Medicine, Tokyo, Japan; 7 Gastroenterology Department, Research Institute Segovia de Arana, Puerta de Hierro University Hospital, Autonomous University of Madrid, Majadahonda, Spain; 8 378609 Digestive Disease Center, Showa University Koto Toyosu Hospital, Koto-ku, Japan; 9 Colorectal Surgery, King’s College Hospital NHS Foundation Trust, London, United Kingdom of Great Britain and Northern Ireland; 10 285211 Gastroenterology, Centro Hospitalar de São João, E.P.E., Porto, Portugal; 11 70918 Glycobiology and Cancer, IPATIMUP, Porto, Portugal; 12 2241 Queen Elizabeth the Queen Mother Hospital, Department of Gastroenterology, East Kent Hospitals University NHS Foundation Trust, Canterbury, United Kingdom of Great Britain and Northern Ireland; 13 16514 Endoscopy Unit, Hospital Universitari Germans Trias i Pujol, Badalona, Spain; 14 16711 Endoscopy Unit, Centro Médico Teknon, Barcelona, Spain; 15 9820 NIHR Nottingham Digestive Diseases Biomedical Research Unit, Nottingham University Hospitals NHS Trust, nottingham, United Kingdom of Great Britain and Northern Ireland; 16 Pathology Department, Marqués de Valdecilla University Hospital, Santander, Spain, Santander, Spain; 17 Clinical and Translational Research in Digestive Diseases, Valdecilla Research Institute (IDIVAL), Gastroenterology and Hepatology Department, Marqués de Valdecilla University Hospital, Santander, Spain; 18 Gastroenterology and Hepatology, Hospital Universitario Ramón y Cajal, Universidad de Alcalá, IRYCIS, Madrid, Spain

**Keywords:** Endoscopy Upper GI Tract, Diagnosis and imaging (inc chromoendoscopy, NBI, iSCAN, FICE, CLE), Endoscopic resection (ESD, EMRc, ...), Endoscopy Lower GI Tract, Endoscopic resection (polypectomy, ESD, EMRc, ...)

## Abstract

**Background and study aims:**

Data on feasibility of endoscopic submucosal dissection (ESD) for treatment of superficial anal squamous cell carcinoma are limited. This study aimed to evaluate outcomes of ESD in this anatomical location.

**Patients and methods:**

This was a multicenter retrospective study including patients who underwent ESD for treatment of superficial ASCC.

**Results:**

Twenty-three patients with superficial ASCC were included. Median lesion size was 24 mm (range, 9–65 mm) and median procedure time was 62 minutes (range, 26–210 minutes). Accuracy of optical diagnosis using Japanese Endoscopic Society Intrapapillary capillary loops (IPCLs) classification to predict final histology was 63.6%. En bloc and R0 resection were achieved in 22 (95.6%) and 18 patients (78.3%), respectively. The curative resection rate was 73.9% (17/23). Three patients received additional complementary treatment. Delayed bleeding was observed in four patients (17.4%), two of whom required endoscopic hemostasis. Anal pain was reported in nine patients (39.1%) and was effectively managed with analgesics. Fecal incontinence and anal stenosis both occurred in one patient during the perioperative period. During median follow-up of 10.1 months (range, 0–69.6 months), no recurrences were observed.

**Conclusions:**

ESD is a feasible and effective treatment for superficial ASCC. Adverse events were successfully managed with medical or endoscopic therapy. ESD should be considered as first-line resection technique to prevent recurrence while preserving anal sphincter function.

## Introduction

Anal squamous cell carcinoma (ASCC) is a rare disease. However, incidence is increasing in Europe, Australia and the United States, with an annual incidence of 0.5 to 2.0 cases per 100,000 inhabitants 1 .

Local excision (LE) is considered the treatment of choice when ASCC is diagnosed at an early stage 2 3 . The main objective is to achieve an “en bloc” resection to facilitate adequate histological analysis, decrease recurrence risk, and preserve sphincter functionality. In this sense, it has been observed that piecemeal resection is associated with high rates of local recurrence, making its use inadvisable 4 5 .

Furthermore, chromoendoscopy and magnifying endoscopy enable clear visualization of superficial microvascular structures to better assess depth of invasion and risk of lymph node metastasis, similar to abnormal intrapapillary capillary loop (IPCL) patterns for superficial esophageal SCC 6 7 . This advanced endoscopic assessment may contribute to better selection of candidates for endoscopic resection, but its diagnostic yield in this location is unknown.

Endoscopic submucosal dissection (ESD) permits “en bloc” resection regardless of tumor size but evidence about its use in ASCC is limited to case reports 6 7 8 9 10 11 12 13 14 . Therefore, we aimed to evaluate safety and long-term efficacy of ESD for ASCC.

## Patients and methods

### Study design

This retrospective study was promoted by the Mucosal Resection and Third Space Endoscopy working group of the Spanish Society of Gastrointestinal Endoscopy. International centers with prospective ESD databases were invited to participate. We included patients who underwent ESD for ASCC at 11 tertiary referral centers in Europe (n = 8) and Japan (n = 3) between November 2015 and August 2024.

The study was approved by the ethics committees for clinical research of the participating centers (institutional review board code: HRYC-DSE-19). Written informed consent was obtained from all patients before inclusion in the prospectively maintained ESD registries. Additional study-specific informed consent was obtained when deemed necessary by local regulations. The study is reported following the STROBE guidelines 15 .

### ESD procedure

Endoscopic submucosal dissection of squamous cell carcinoma of the anal canal using conventional ESD technique.Video 1

**Fig. 1:**
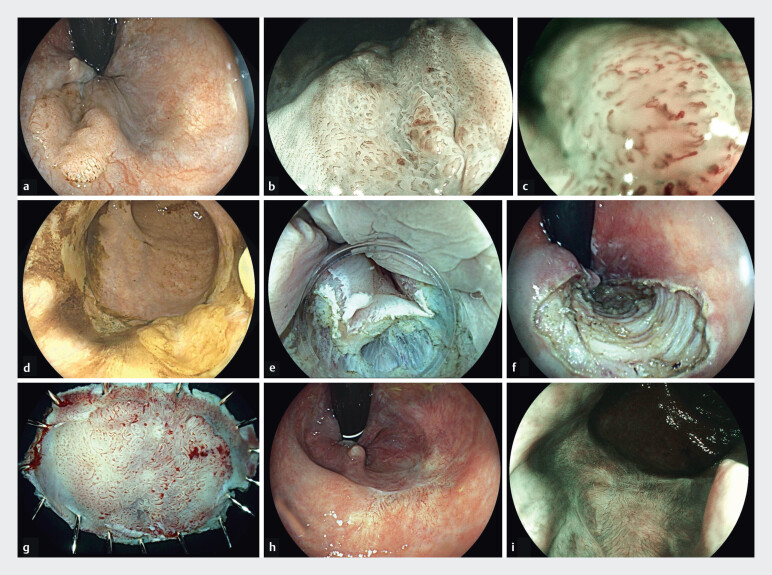
Squamous cell carcinoma of the anal canal. **a** White light (WL) image showing 20 × 12 mm flat elevated lesion over the dentate line. **b** Blue light imaging (BLI) with a well-defined lesion border. **c** Magnified BLI images showing dilation, weaving, and elongation of IPCL-like micro vessels. **d** Lugol stain-negative lesion with clearly defined lesion margins. **e** Incision in the anal canal with submucosal exposure. **f** Endoscopic submucosal dissection scar **g** Specimen resected “en bloc”. **h, i** Three months of follow-up (WL & BLI).

**Fig. 2:**
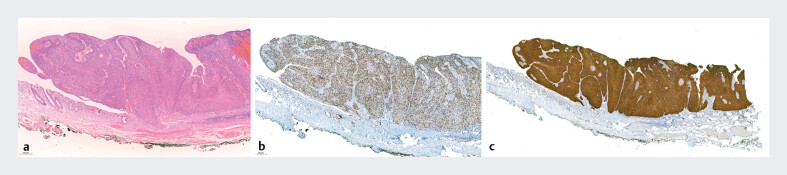
Histological evaluation. **a** Hematoxylin and eosin staining (x5). **b** Immunohistochemical staining for Ki67 throughout mucosal thickness (x5). **c** High p16 staining showed a diffuse-positive pattern. throughout mucosal thickness (x2).

ESD technique and materials were at the discretion of each endoscopist ( Fig. 1 , Fig. 2 , Video 1 ). Decisions about ESD for LE and/or need for complementary treatment (surgery, radiotherapy [RT] and/or chemotherapy) were discussed at the local multidisciplinary oncologic committees of each institution. Pretreatment assessment studies and follow-up were carried out following oncological clinical guidelines at each center.

### Definitions and endpoints

Primary endpoints were the curative, “en bloc” and R0 resection rates. Curative resection was defined as free margin resection (R0) and absence of high-risk histologic criteria for lymph node metastasis (lymphovascular invasion, submucosal invasion, or poorly differentiated tumor). Secondary endpoints were the adverse event (AE) rate, use of chromoendoscopy and magnification for pretreatment assessment, and analysis of overall and progression-free survival. Delayed bleeding was defined by at least one of the following criteria: 1) rectal bleeding or hematochezia associated with clinical signs of bleeding after 24 hours of the procedure; 2) a drop-in hemoglobin ≥ 2 g/dL related to ESD (> 24 hours after the procedure); and 3) need for therapeutic intervention such as endoscopic hemostasis, surgery, or blood transfusion due to bleeding (24 hours after the procedure). AE severity was graded using the Adverse events in GastRointEstinal Endoscopy (AGREE) classification 16 .

### Statistical analysis

Data analysis was performed using IBM SPSS Statistics, Version 20.0 (Chicago, Illinois, United States). The Wilson method was used to calculate 95% confidence intervals (CIs). Categorical variables were expressed as absolute frequencies and percentages. Continuous variables were expressed as medians with ranges. Missing values are described in Table 1 and Table 2 .

**Table:** **Table 1** Study population and patient characteristics.

Characteristic	Value
Number of patients	23
Age (years), median (range)	65 (42–85)
Female, n (%)	17 (73.9)
Active smoker, n (%)	4 (17.4)
History of previous SCC in other location, n (%)	
Gynecological	2 (8.7)
Laryngeal	1 (4.3)
HIV, n (%)	2 (8.7)
HPV, n (%)	
HPV negative	11 (47.8)
HPV genotype 16	8 (34.8)
Missing	4 (17.4)
MSM/WSW, n (%)	
No	12 (52.1)
Yes	3 (13)
Missing	8 (34.8)
ASA functional status, n (%)	
I	11 (47.8)
II	7 (30.4)
III	5 (21.7)
Charlson Comorbidity Index, median (range)	2 (0–7)
Missing	2
Patient fit for radical surgery, n (%)	20 (87)
Missing	3 (13)
Anticoagulant therapy, n (%)	0 (0)
Antiplatelets, n (%)	
No	19 (82.6)
Aspirin	3 (13.1)
Clopidogrel	1 (4.3)
ASA, American Society of Anesthesiologists; HPV, human papillomavirus; MSM, men who have sex with men; SCC, squamous cell carcinoma; SCC, squamous cell carcinoma; SD, standard deviation; WSW, women who have sex with women.	

**Table:** **Table 2** Lesion characteristics and endoscopic submucosal dissection procedure details.

Lesion characteristics	Value
Preprocedure biopsy findings, n (%)	
Not performed	8 (34.8)
Low-grade dysplasia	4 (17.4)
High-grade dysplasia	8 (34.8)
Intramucosal carcinoma	3 (13)
JES IPCL Classification, n (%)	
A	2 (8.7)
B1	5 (21.7)
B2	4 (17.4)
Not assessed	12 (52.1)
Morphology (Paris classification), n (%)	
Is	4 (17.4)
IIa	12 (52.1)
IIb	4 (17.4)
Is+IIb	1 (4.3)
IIa+IIc	2 (8.7)
**ESD procedure**	
Endoscopist previous ESD experience, n (%)	
< 50 cases	1 (4.3)
50–100 cases	3 (13)
> 100 cases	19 (82.6)
Sedation, n (%)	
General Anesthesia	12 (52.2)
Deep sedation	7 (30.4)
Conscient sedation	4 (17.4)
ESD strategy, n (%)	
Circumferential	16 (69.6)
Pocket	2 (8.7)
Tunnel	5 (21.7)
Traction, n (%)	
No	22 (95.7)
Yes (external forceps)	1 (4.3)
Fibrosis, n (%)	
F0	8 (34.8)
F1	10 (43.5)
F2	1 (4.3)
Missing	4 (17.4)
Injection of local anesthetic, n (%)	
No	17 (73.9)
Yes	6 (26.1)
Type of knife used, n (%)	
Needle knife	19 (82.6)
Needle knife + isolated tip knife	1 (4.3)
Scissors type	1 (4.3)
Bipolar knife	2 (8.7)
ESD, endoscopic submucosal dissection; JES IPCL, Japan Esophageal Society Intrapapillary Capillary Loop Classification.	

## Results

The study included a total of 23 patients (17 women [73.9%]; median age 65 years (range: 42–85) with ASCC. Eight of 19 (42.1%) were HPV-16-related lesions and only two (8.7%) presented HIV coinfection. Two patients presented SCC HPV-related tumors in other locations: one had a history of laryngeal (cT2N0M0) and vulvar (pT1N0M0) SCC treated with RT and LE, respectively, and the other had a synchronous vulvar lesion (pT1N0M0) treated by LE. Additional baseline characteristics are shown in Table 1 .

### Pretreatment assessment

Pretreatment workup to rule out locoregional and/or metastatic disease was heterogeneous: four of 23 patients (17.4%) underwent both thoraco-abdomino-pelvic computed tomography (CT) scan and pelvic magnetic resonance imaging (MRI), eight (34.78%) had a CT scan and two (8.7%) a MRI. No positron emission tomography (PET)-CT scans were performed and only one patient from the first group above received an endoscopic ultrasound examination.

High-quality endoscopic assessment (high definition and virtual chromoendoscopy) was performed in all of them. Lugol stain was used in seven patients (30.4%) to clearly delineate tumor margins. Optical magnification was available in 15 (65.2%) and the Japan Esophageal Society (JES) intrapapillary capillary loop (IPCL) Classification for SCC was used in 11 (47.8%) with a diagnostic accuracy to predict final histology of seven of 11 (63.6%, 95% CI 35%-84%) (3/11 underestimated; 1/11 overestimated). Previous biopsies were taken in 15 patients (65.2%), with final histology concordance in 10 of 15 (66.7%, 95% CI 42%-85%) (5 of 15 underestimated the final histology).

Regarding anatomical ASCC location, 14 lesions (60.9%) were located in the anal canal with rectal extension, seven (30.4%) in the anal canal extending to both the rectum and anal margin, one (4.3%) was limited to the anal canal, and one (4.3%) was located in the anal canal with extension to the anal margin.

### ESD procedure

Twelve procedures (52.2%) were performed under general anesthesia. Initial circumferential cutting was the most common strategy (69.6%) and the needle-type knives were the preferred ones (82.6%) ( Table 2 ). Six patients (26.1%) had topical anesthetic injection during ESD to prevent postoperative anal pain. One ESD case was performed after recurrence of a previous surgical LE.

### Resection outcomes

En bloc and R0 resection rates were 95.6% (22 of 23, 95% CI 79%-99%) and 78.3% (18 of 23, 95% CI 58%-90%), respectively. Curative resection rate was slightly lower (73.9%, 17 of 23, 95% CI 54%-88%). Reasons for non-curative ESD were: three carcinomas with submucosal invasion, one positive vertical margin with lymphovascular invasion, and two positive horizontal margin (1 with free margin < 1 mm and 1 affected with dysplasia). Additional treatment was performed in three of six non-curative ESDs. Median follow-up for these patients receiving adjuvant treatment was 17.9 months (range, 10.9–46.2). Median interval between ESD and adjuvant treatment was 2.63 months (range, 2–4). Details of non-curative resections and further management are provided in Table 3 .

**Table:** **Table 3** Summary of cases treated with endoscopic submucosal dissection.

Age	Gender	HPV/genotype	Paris	JES	Previous biopsy	Size (mm)	Final histology	Depth (micras)	LV invasion	Differentiation	Lateral/vertical margins	Additional treatment	Follow-up (months)
85	M	No	IIa+IIc	-	No	15	Invasive carcinoma	4150	Yes	G3	+/-	No	10.9
81	F	Unknown	IIa	B1	LGD	55	Invasive carcinoma	400	No	G1	+/+	CRT	46.2
74	F	No	Is	-	HGD	40	HGD	-	No	G1	+/-	No	36.1
73	F	Yes/-	IIa	A	No	65	HGD	-	No	-	-/-	No	11.2
72	F	Unknown	IIa	B1	Carcinoma	20	Intramucosal	-	No	G1	-/+	No	8
70	F	No	Is	-	No	50	Intramucosal	-	No	G1	+/-	No	70
70	F	Yes/16	IIb	-	HGD	25	HGD	-	No	-	-/-	No	53
69	F	Yes/16	Is	-	HGD	9	Invasive carcinoma	3000	No	G1	-/-	No	29
67	M	No	IIa	-	HGD	30	HGD	-	No	-	-/-	No	13
67	F	No	IIa	-	No	20	Invasive carcinoma	<200	No	G1	-/-	RT	11
65	F	No	IIa	-	No	15	HGD	-	No	G1	-/-	No	63
60	F	No	Is+IIb	B2	Carcinoma	30	Intramucosal	-	Yes	G1	-/-	No	7.2
59	M	Yes/16	IIb	-	LGD	20	HGD	-	No	-	-/-	No	4.6
59	F	Yes/16	IIa	B2	LGD	25	HGD	-	No	G1	-/-	No	7.4
56	F	Yes/-	IIb	-	HGD	20	HGD	-	No	-	-/-	No	2.8
56	M	Unknown	Is	B2	Carcinoma	12	Intramucosal	-	Yes	G1	+/+	CRT	24.7
52	F	Unknown	IIa+IIc	-	No	25	Invasive carcinoma	3000	Yes	G1	-/-	No	0
46	F	No	IIa	B1	LGD	20	LGD	-	No	G1	-/-	No	9.4
42	F	Yes/16	IIa	A	No	15	LGD	-	No	G1	-/-	No	1
62	M	Yes/-	IIa	B1	No	60	HGD	-	No	G1	-/-	No	2.5
63	M	No	IIa	B2	HGD	40	HGD	-	No	G1	+/-	No	1
72	F	No	IIa	-	HGD	20	Intramucosal	-	No	G1	-/-	No	1.3
43*	F	No	IIb	B1	HGD	20	HGD	-	No	G1	-/-	No	1.1
CRT, chemoradiotherapy; HGD, high-grade dysplasia; HPV, human papillomavirus; JES IPCL, Japan Esophageal Society Intrapapillary Capillary Loop Classification; LGD, low-grade dysplasia; LV, lymphovascular invasion; RT, radiotherapy. *Endoscopic submucosal dissection for managing recurrence following surgical local excision.													

### Adverse events

There were no clinically relevant bleeds (those not controlled with hemostatic forceps or that led to suspension of the procedure, surgery, or radiological intervention). Delayed bleeding occurred in four patients (17.4%, 95% CI 8%–28%): three had previous intraprocedural bleeding, (two were managed conservatively, and one required endoscopic hemostasis) while one experienced a de novo bleed 9 days after ESD in a patient without antithrombotic agents, requiring readmission and endoscopic treatment. Postprocedural anal pain occurred in nine patients (39.1%, 95% CI 22%–59%): six (26.1%) were managed with non-opioids, two (8.7%) with minor, and one (4.3%) with major opioids. Only one of the three patients who underwent postoperative RT experienced anal pain as a complication which was also treated with oral analgesics. One patient reported fecal incontinence (4.3%, 95% CI 1%–21%) during the perioperative period, which gradually resolved. One other patient developed anal stenosis 2 months after ESD, which was successfully resolved after a single session of digital plus bougie dilation and subsequent topical steroid treatment. None of the patients receiving adjuvant therapy developed stenosis ( Table 4 ).

**Table:** **Table 4** Outcomes and adverse events.

Outcomes	
En bloc resection, n (%)	22 (95.6)
R0 resection achieved, n (%)	18 (78.3)
Specimen major axis (mm), median (range)	40 (15–75)
Specimen minor axis (mm), median (range)	24 (13–60)
Curative resection, n (%)	17 (73.9)
Procedure time (min), median (range)	62.5 (26–210)
Final histology, n (%)	
Low–grade dysplasia	2 (8.7)
High–grade dysplasia	11 (47.8)
Intramucosal carcinoma	5 (21.7)
Invasive carcinoma	5 (21.7)
**Adverse events**	
Anal pain, n (%)	9 (39.1)
Grade I	2 (9.5)
Grade II	3 (14.3)
Delayed bleeding, n (%)	4 (17.4)
Grade I	1 (4.3)
Grade II	1 (4.3)
Grade IIIa	2 (8.7)
Fecal incontinence, n (%)	1 (4.3)
Grade I	1 (4.3)
Stenosis, n (%)	1 (4.3)
Grade I	1 (4.3)

### Follow-up

Seventeen of 23 patients underwent follow-up endoscopy (73.9%); one of them was also followed with a CT scan and pelvic MRI. Two of 23 (8.7%) underwent CT scan and MRI exclusively, and one (4.3%) MRI and PET scan. All patients who underwent complementary treatment after ESD were followed with radiological imaging tests. Median follow-up was 10.1 months (range, 0–69.6). There were neither deaths nor tumor progression at the end of follow-up.

## Discussion

Results from this multicenter study demonstrate that ESD is an effective and safe technique for treatment of superficial ASCC. Although endoscopic techniques have evolved significantly in recent years, their roles in current management of superficial ASCC—both for diagnosis and treatment—are not yet well-established, despite improvements in safety and technology 2 3 .

LE is an effective treatment for T1N0M0 and some selected T2N0M0 ASCC cases, with recurrence rates similar to chemoradiotherapy (CRT) but with a lower rate of late AEs (hematologic or gastrointestinal toxicity, dermatitis, proctitis, anal or vaginal stenosis, incontinence, fecal urgency, or sexual dysfunction). Moreover, by choosing LE as the first treatment option, RT and/or CRT is saved for hypothetical locoregional recurrences and/or treatment of other pelvic malignancies 4 17 . However, because risk of locoregional recurrence after LE is still not well defined, need for adjuvant treatment should be evaluated on a case-by-case basis, and close follow-up should always be performed. Radical surgery in T1–2N0M0 is currently reserved as salvage treatment after progression to RT/CRT or LE 3 .

Despite good accessibility, sinuous morphology and nerve sensitivity, together with high vascularization of the hemorrhoidal plexus can make ESD challenging in this location. Nonetheless, en bloc, R0, and curative resection rates in our series were high at 95.7%, 78.3% and 73.9% respectively, supporting use of ESD as a first-line full-biopsy resection treatment. No local recurrences were detected during surveillance after ESD, which outperforms recurrence rates reported after surgical excision (9%–63%) 4 18 , and other less invasive topical (trichloroacetic acid, imiquimod, 5-fluorouracil) and ablative techniques 19 . Furthermore, treating ASCC with ESD theoretically eliminates need for reintervention and subsequent increased associated risk of stricture and/or fecal incontinence.

In consonance with what was previously reported, the majority of cases in our series were women (80%), and were related to HPV-16, for which gynecological evaluation to rule out concomitant lesions is highly recommended. Special attention should be paid during routine colonoscopies in patients with other well-known associated risk factors for ASCC, such as smoking, HIV, receptive anal sex, cervical or perineal cancer, or immunosuppression 3 . Involvement of other areas such as the oropharyngeal region should be individually assessed.

Pretreatment staging in our series was heterogeneous, mainly based on MRI and CT to exclude locoregional and distant disease. Endoscopic ultrasound (EUS)-guided fine needle aspiration was not performed in any patient because biopsy was not deemed necessary to clarify the nature of suspicious lymphadenopathy, either prior to ESD or during follow-up. Although use of EUS to assess tumor invasion depth remains controversial, it may be considered in carefully selected cases. All patients underwent high-quality (high-definition and chromoendoscopy) endoscopic assessment. Use of Lugol stain in this series was infrequent (30.4%). However, when used either alone or in combination with acetic acid, it may improve lesion delineation, aid in detection of small lesions that are not visible to the naked eye, and enhance accuracy of targeted biopsies. Although vital stains have demonstrated good sensitivity (80%–90%), false negatives may still occur, especially in small lesions where iodine absorption is insufficient. Virtual chromoendoscopy techniques offer slightly higher sensitivity (90%–95%) and are particularly useful for identifying small, superficial, or anatomically challenging lesions. For these reasons, both methods are regarded as complementary tools that can further improve early detection of SCC throughout the gastrointestinal tract.

Accuracy of JES IPCL Classification for predicting final histology was moderate (63.6%), closely matching the 66.7% accuracy obtained from prior biopsies. Although this classification has not been formally validated for this anatomical location, by analogy with findings in the esophagus, presence of aberrant microvessels—characterized by dilation, tortuosity, caliber changes, and irregular shapes—may indicate increased risk of deep tumor invasion. Interestingly, two cases were initially classified as JES type A, and no confirmatory biopsies were performed. Although current ASCC clinical guidelines recommend performing a diagnostic biopsy, eight of 23 patients (34.8%) did not undergo one prior to ESD due to concerns that biopsy-related fibrosis in flat lesions might hinder successful resection. In such instances, the ESD specimen was regarded as a high-quality biopsy in itself. Given the lower rate of early SCC detection in Western countries, it is crucial to assess endoscopist experience and proficiency with the JES classification before relying on it for diagnostic purposes. These findings highlight the added value of optical endoscopic diagnosis performed by trained endoscopists, which provides complementary information in pretreatment staging and should always be considered.

AEs were medically managed with no protocol deviation. Most of the lesions had rectal extension, which implied performing ESD through the hemorrhoidal plexus and increased risk of bleeding 20 . However, delayed bleeding occurred in only four patients after ESD; two were managed conservatively, whereas the other two required endoscopic intervention. Anal pain occurred in 39.1% of patients, all of whom were successfully managed with perioperative analgesia and the pain gradually disappeared. Post-ESD anal pain is a relevant clinical concern influenced by several factors, including individual sensitivity, dissection technique employed, and thermal damage to the muscularis propria. In this series, only six patients (26.1%) received topical anesthetic injections for pain prevention; among them, three (50%) still experienced postoperative pain, which was managed with analgesics. Of the 17 patients (73.9%) who did not receive any form of preventive treatment, six (35.3%) reported postoperative pain, all of whom also responded well to analgesic therapy. These findings suggest that routine preventive analgesia may not be necessary as a standard approach and should instead be tailored on a case-by-case basis.

Regarding worrying risk of stricture or fecal incontinence, each occurred only temporarily in one patient, including those who required complementary treatment. Although use of steroids may help prevent rectal stenosis, their efficacy in this anatomical location remains unclear. Therefore, their use may be considered selectively in high-risk cases.

Fecal incontinence is a potential complication that must be anticipated. Thorough evaluation of patient medical history is essential, and in cases of uncertainty, anorectal manometry should be considered to assess sphincter integrity and tone. Risk factors such as history of instrumental vaginal delivery, previous anal surgery, advanced age, neurological disorders, or diabetes mellitus may impair sphincter function and increase risk of incontinence. In addition, procedure factors related to ESD—such as involvement of the internal anal sphincter, resection of more than 50% of the anorectal circumference, or extension to the dentate line—can further contribute to development of fecal incontinence. Another possible contributing factor is reduced rectal compliance due to fibrosis, which may lead to urgency-related incontinence. To minimize risk of these complications, a multimodal preventive strategy is recommended. This may include local steroid injections, limited or targeted dissections, a high level of technical expertise in ESD, and use of non-aggressive electrosurgical settings. These measures are essential to reduce tissue injury, reduce risk of fibrosis, and preserve anorectal function.

The main strengths of our study are that it represents the largest ESD cohort of superficial ASCC involving 11 referral centers and that the data are derived from prospectively collected databases. However, we acknowledge that our study has limitations. First is the limited sample size and its retrospective design. Second, the institutions that participated have extensive experience in ESD, and therefore, results should be interpreted with caution. Finally, the follow-up period was short and staging and surveillance methods after ESD were heterogeneous, and therefore, long-term conclusions cannot be drawn.

## Conclusions

In conclusion, ESD seems an effective and safe resection technique for treatment of superficial ASCC and should be considered as a first-line therapeutic option. In expert hands, “en bloc” resection rates are high, reducing recurrences and risks associated with reintervention. Furthermore, in cases of non-curative ESD, adjuvant treatments can be performed maintaining sphincter functionality and quality of life of these patients. Larger prospective comparative studies with longer follow-up are needed to assess the role of ESD and to compare it with other modalities of treatment in this clinical setting.
